# Dual-Sizing Effects of Carbon Fiber on the Thermal, Mechanical, and Impact Properties of Carbon Fiber/ABS Composites

**DOI:** 10.3390/polym13142298

**Published:** 2021-07-13

**Authors:** Daekyun Hwang, Seung Goo Lee, Donghwan Cho

**Affiliations:** 1 Department of Polymer Science and Engineering, Kumoh National Institute of Technology, Gumi 39177, Gyeongbuk, Korea; ghkdeorbs12@naver.com; 2 Department of Organic Materials Engineering, Chungnam National University, Deajeon 34134, Korea; lsgoo@cnu.ac.kr

**Keywords:** carbon fiber-reinforced composites, ABS resin, extrusion/injection, properties, dual-sizing

## Abstract

Dual-sizing effects with either epoxy or polyurethane (PU) on the thermal, mechanical, and impact properties of carbon fiber/acrylonitrile-butadiene-styrene (ABS) composites produced by extrusion and injection molding processes were investigated. The heat deflection temperature, dynamic mechanical, tensile, flexural, and impact properties of the composites reinforced with either (epoxy + epoxy) or (epoxy + PU) dual-sized carbon fiber were higher than those commercially single-sized with epoxy. The result indicated that the dual-sized carbon fiber significantly contributed not only to improving the heat deflection temperature and the storage modulus, but also to improving the tensile, flexural, and impact properties of carbon fiber/ABS composites. The highest improvement of the composite properties was obtained from the composite with (epoxy + PU) dual-sized carbon fiber. The improvement of the mechanical and impact properties was explained by the enhanced interfacial bonding between carbon fiber and ABS matrix and by the length distribution analysis of carbon fibers present in the resulting composites. The fiber–matrix interfacial behavior was qualitatively well-supported in terms of fiber pull-out, fiber breaking pattern, and debonding gaps between the fiber and the matrix, as observed from the fracture surface topography. This study revealed that the properties of carbon fiber/ABS composites prepared by extrusion and injection molding processes were improved by dual-sizing carbon fiber, which was performed after a commercial epoxy sizing process, and further improved by using PU as an additional sizing material.

## 1. Introduction

Recently, it has been known that the use of carbon fiber-reinforced plastics (CFRP) is one of the promising approaches not only to reduce the weight of automobile parts, but also to increase the properties and performances of many industrial parts by replacing conventional metal-based and glass fiber-reinforced plastic materials [1,2,3].

A composite material is a combination of two or more individual materials with different constitution and composition. Composite materials have advantages of lightweight, corrosion resistance, and high mechanical strength and modulus. Moreover, CFRP provides good damping characteristics and high fatigue resistance. Accordingly, CFRP has been widely used as structure material for a long period of time. To date, CFRP has been extensively used in parts applications such as aerospace, aircraft, automobile, marine, building, electronics, sports/leisure, and oil extraction industries. The relationship among high mechanical property, fatigue resistance, corrosive resistances, thermal properties, etc. has been importantly considered in relevant applications of CFRP [4,5,6,7,8].

In addition, CFRP has been increasingly used in civil engineering due to its high specific mechanical properties and corrosion resistance. Considering the long service life of civil engineering structures, the mechanical properties of CFRP are fundamentally determined by the tensile and flexural strength and modulus, as well as the interfacial bonding strength. Good interfacial bonding can effectively transfer external stress from the matrix to the reinforcing fiber, enhancing the ultimate strength [7].

More particularly, the fatigue resistance of CFRP becomes the important criterion to evaluate the security of a structure in the long-term service of the related structures. Generally, CFRP exhibits excellent fatigue performance when compared to metallic construction materials. The fatigue performance depends on the interfacial bonding between fiber and matrix and the mode of applied load [4]. Moreover, laminated CFRP is increasingly used to replace conventional aluminum parts in the aircraft industry due to its lightweight structure [5]. In addition, to achieve the balance of cost and performance with fiber-reinforced composites, a kind of carbon fiber/glass fiber-reinforced polymer hybrid composite can be produced by pultrusion technology for new applications [4]. They exhibit the advantages of high mechanical and corrosion resistance of carbon fiber and low price and high deformation of glass fiber.

Thermoplastic matrix-based CFRP has been increasingly researched and developed in academia and industry in recent years [9,10,11,12,13]. This is because thermoplastic CFRP has advantages such as fast processing without cure reaction, mass productivity, fracture toughness, re-moldability, and recyclability over thermosetting CFRP [14], although thermosetting CFRP has received much more attention than thermoplastic CFRP for a long period of time since carbon fiber was developed in the 1960s [15,16]. However, thermoplastic CFRP has some disadvantages, such as difficulties in resin impregnation, due to high melt viscosity and low resin flow, and mechanical properties due to weak bonding at the interface between the carbon fiber and polymer matrix over thermosetting CFRP [17]. Hence, extrusion and injection molding processes have been most frequently used to prepare thermoplastic CFRP using chopped carbon fibers [18,19].

In polyacrylonitrile-based carbon fiber manufacturing, stabilization, carbonization, graphitization (optional), oxidative surface treatment, sizing, and drying processes are carried out necessarily and proprietarily via continuous routes [20,21,22]. Conventionally, surface treatment can be done by oxidizing carbon fiber with an electrolytic medium, followed by sizing process, commercially with epoxy at less than 2% [23,24]. The purpose of sizing is to protect the carbon fiber surface, to improve the handleability, and further to improve the fiber–matrix interfacial bonding of a composite material [20]. The interfacial bonding between carbon fiber and thermoplastic matrix is relatively weak. The increase of the interfacial bonding may result in a property improvement of resulting composites because external loads can be more efficiently transferred from the matrix phase to the fiber phase [25,26]. Hence, many papers have dealt with the increased mechanical properties of thermoplastic CFRP by improving the interfacial bonding [17,27]. Sizing agents may influence the interfacial bonding between the fiber and the matrix, depending on polymer type, their characteristic, and compatibility with the matrix resin. Many papers have been reported on the carbon fibers sized with epoxy as sizing agent and their composites [28,29,30,31,32,33]. Several papers have dealt with other sizing agents beside epoxy [34,35,36].

Ozkan et al. [32] studied the effect of the type and concentration of sizing agents (epoxy/phenoxy, polyimide, and phenoxy) on the mechanical and electrical properties of carbon fiber/polycarbonate composites prepared by extrusion and injection molding processes. They found that the tensile strength and modulus of sized carbon fiber-reinforced polycarbonate composites were higher than those of the unsized counterpart. The effect of the sizing material level on the tensile properties of composites depended on the sizing material type. They reported that sized carbon fiber-reinforced polycarbonate composites had higher electrical conductivity than the unsized counterpart. Moreover, phenoxy-sized composite exhibited the highest electrical conductivity compared to the composites sized with epoxy/phenoxy and polyimide. The increased interfacial interactions between phenoxy-sized carbon fiber and polycarbonate matrix were responsible for the increased mechanical property and electrical conductivity of resulting composites, as found from the fracture surface analysis. Karsli et al. [35] reported how sizing agents, such as polyurethane, polyamide, polyimide, and phenoxy, influenced the mechanical properties of carbon fiber/polyamide 6,6 composites.

From an industrial viewpoint, acrylonitrile-butadiene-styrene (ABS) is one of the most important thermoplastic resins with a combination of good mechanical, thermal, and impact properties and processability, depending on the chemical composition of acrylonitrile, butadiene, and styrene components therein [37,38,39]. It has been widely used in many industrial parts, such as automobiles, appliances, electronic housings, etc. Several papers on CFRP with ABS matrix have been reported [39,40]. However, carbon fiber/ABS composites dealing with dual-sized carbon fiber have not been reported yet. Here, “dual-sizing” refers to commercial sizing plus additional sizing. Therefore, dual-sizing effects on the thermal, mechanical, and impact properties of carbon fiber/ABS composites are worth studying.

Consequently, the objective of the present study is to explore how dual-sizing, which was carried out additionally with commercially epoxy-sized carbon fiber, influences the thermal, mechanical, and impact properties of carbon fiber/ABS composites. For this, firstly, three different types of carbon fiber/ABS composites consisting of (epoxy) single-sized and either (epoxy + epoxy) or (epoxy + polyurethane) dual-sized carbon fibers were prepared via twin-screw extrusion and injection molding processes. Secondly, the fiber surface, fiber length distribution, thermal stability, heat deflection temperature, dynamic mechanical, tensile, flexural, and impact properties and the fracture surface behavior were extensively investigated and compared.

## 2. Materials and Methods

### 2.1. Materials

Chopped carbon fibers of 6 mm long in average, which were kindly supplied from ACE C&TECH Co., Ltd., Youngjoo, Gyeongbuk, Korea, were utilized for twin-screw extrusion process. The chopped carbon fibers were produced through proprietary sizing process with either epoxy or polyurethane (PU), which was additionally performed with commercial PAN-based carbon fiber (12K, epoxy-sized, T-700 grade, Toray Advanced Materials Co., Ltd., Gumi, Gyeongbuk, Korea). Two different types of sizing materials (epoxy and PU) were additionally applied to commercially epoxy-sized carbon fiber. Hereinafter, commercial epoxy sizing is referred to as ‘single-sizing’, whereas additional sizing performed after single-sizing is referred to as ‘dual-sizing’. Dual-sizing with epoxy on the commercially epoxy-sized carbon fiber is referred to as ‘(epoxy + epoxy)’, whereas dual-sizing with PU on the commercially epoxy-sized carbon fiber is referred to as ‘(epoxy + PU)’. The dual-sizing process was proprietarily carried out with either epoxy or PU for efficiently chopping carbon fibers. The concentration of additional sizing materials (epoxy and PU) dispersed in water was approximately 2 wt%.

ABS resin (Model ABS740), which was manufactured by Kumho Petrochemical Co., Ltd., Seoul, Korea, was supplied in pellet form and used as thermoplastic matrix of CFRP. According to the manufacturer’s information, the compositions of acrylonitrile, butadiene, and styrene in ABS resin are approximately 20%~30%, 10%~30%, and 50%~65%, respectively. The density is 1.04 g/cm^3^ and the melt flow index (220 °C, 10 kg) is 25 g/10 min. It was revealed that Model ABS740 exhibited higher impact resistance than other ABS resin grades commercialized by the company. All carbon fibers and ABS pellets were sufficiently dried at 80 °C for 12 h in a convection oven prior to use.

### 2.2. Preparation of Carbon Fiber/ABS Pellets and the Composites

**Figure 1:**
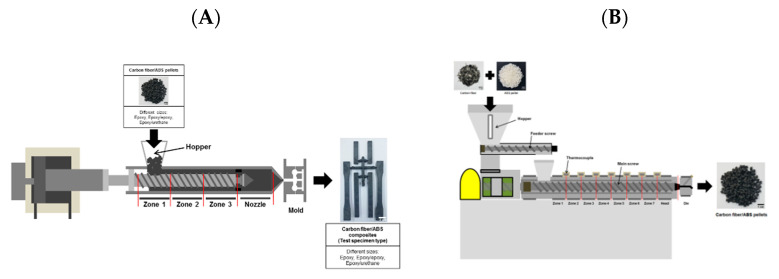
Schematics of extrusion process for preparing (**A**) carbon fiber/ABS pellets and (**B**) injection molding process for preparing carbon fiber/ABS composites.

To prepare carbon fiber/ABS pellets using chopped carbon fibers with different sizing materials, twin-screw extrusion process was performed. A modular intermeshing co-rotating-type twin-screw extruder (BT-30-S2-421, LG Machinery Co., Ltd., Daejeon, Korea) with a screw diameter of 30 mm and L/D ratio of 42 was used. Chopped carbon fibers and ABS pellets were pre-mixed in a plastic bag, then regularly fed through the hopper. The contents of chopped carbon fibers were fixed to 30% by weight throughout the work. The processing temperature was varied in the range of 160~225 °C, depending on the barrel zone, head, and extrusion die. The screw speed was 80 rpm. The feeding rate was 9 kg/h. Dual-sized carbon fiber/ABS pellets approximately 2–3 mm long were prepared by cooling them in a water-bath and consecutively by pelletizing. Single-sized carbon fiber/ABS pellets were also prepared for comparison. The extrusion process for preparing carbon fiber/ABS pellets was illustrated in Figure 1A.

After drying the pellets at 80 °C for 12 h, the injection molding process was performed with the pellets using an injection molding machine (Model PRO-WD 80, Dongshin Hydraulics Co. Ltd., Changwon, Korea) for producing carbon fiber/ABS composites. The barrel temperatures were varied in the range of 230–250 °C. The mold temperature was 80 °C. The carbon fiber contents were fixed to 30% by weight. Dual-sized carbon fiber/ABS composites were prepared. Single-sized carbon fiber/ABS composites were also prepared under the same processing condition for comparison. The injection molding process for preparing carbon fiber/ABS composites is illustrated in Figure 1B. The injection-molded composites were used as specimens for heat deflection temperature, dynamic mechanical, tensile, flexural, and impact tests.

### 2.3. Characterization

#### 2.3.1. Scanning Electron Microscopic Analysis

Scanning electron microscopy (SEM, JSM 6380, JEOL, Tokyo, Japan) was performed to observe the topography of single-sized and dual-sized carbon fibers and the fracture surfaces of carbon fiber/ABS composites after impact testing. Prior to SEM observation, all samples were uniformly coated with platinum for 3 min by a sputtering method. The acceleration voltage used was 15 keV and SEM micrographs were obtained with a secondary electron image (SEI) mode.

#### 2.3.2. X-ray Photoelectron Spectroscopy

X-ray photoelectron spectroscopy (XPS, Multilab-2000, ThermoFisher Scientific, Waltham, MA, USA) was performed to measure the chemical compositions of single-sized and dual-sized carbon fiber surfaces. The X-ray source was Al-K_α_ radiation. The lens mode and the energy step size were LAXPS and 1.0 eV, respectively.

#### 2.3.3. Thermogravimetric Analysis

The thermal stability of neat ABS and single-sized and dual-sized carbon fiber/ABS composites was examined up to about 800 °C, purging a nitrogen gas by a thermogravimetric analyzer (TGA Q500, TA Instruments, New Castle, DE, USA). For each measurement, the sample of about 10~12 mg was placed in an alumina pan. The heating rate was 20 °C/min.

#### 2.3.4. Fiber Length Distribution Analysis

The carbon fiber length distribution of single-sized and dual-sized carbon fiber/ABS composites was measured by using a co-axial optical microscope (HD-200, LeeTech Co., Seoul, Korea). Each composite sample was placed in a vial (50 mL) containing acetone for a sufficient duration to dissolve the ABS matrix therein. Then, the individual carbon fiber filaments separated from each composite sample were placed and spread on a glass slide using a dropper. The length of fiber filaments was measured at the magnification of 600×. The fiber length distribution histogram was given, based on the lengths of 650 fiber filaments obtained from each composite sample.

#### 2.3.5. Heat Deflection Temperature Measurement

The heat deflection temperature (HDT) of neat ABS and single-sized and dual-sized carbon fiber/ABS composites was measured using an HDT tester (Tinius Olsen, Model 603, Horsham, PA, USA) according to the ASTM D648 standard. All measurements were performed in a silicone oil bath heated with the heating rate of 120 °C/h after each specimen was soaked in the bath for 3–5 min. The HDT value of each specimen was recorded when the deflection of 0.254 mm in the specimen occurred during measurement. The dimensions of the bar-shaped specimen were 125 mm × 12.5 mm × 3 mm. The heating rate was 2 °C/min. Each measurement was carried out under the three-point bending load of 0.455 MPa. The average HDT value of each sample was obtained from 3 specimens.

#### 2.3.6. Dynamic Mechanical Analysis

The storage modulus and tan δ of neat ABS and single-sized and dual-sized carbon fiber/ABS composites were examined with the heating rate of 2 °C/min in air by using a dynamic mechanical analyzer (DMA Q800, TA Instruments, New Castle, DE, USA). A dual cantilever mode with a drive clamp and a fixed clamp was used throughout DMA. The measuring temperature range was from ambient temperature to 160 °C. The oscillation amplitude was 10 μm and the frequency was 1 Hz. The dimensions of the bar-shaped specimen were 63.5 mm × 12.5 mm × 3 mm.

#### 2.3.7. Tensile Test

Tensile tests of neat ABS and single-sized and dual-sized carbon fiber/ABS composites were performed at ambient temperature in accordance with the ASTM D648 standard using a universal testing machine (UTM, Shimadzu JP, AG-50kNX, Kyoto, Japan). The dimensions of dog-bone shaped specimen were 165 mm × 12.5 mm × 3 mm. The gage length was 100 mm. A load cell of 50 kN and the crosshead speed of 5 mm/min were used. The average values of tensile modulus and strength of each sample were obtained from 10 specimens.

#### 2.3.8. Flexural Test

Three-point flexural tests of neat ABS and single-sized and dual-sized carbon fiber/ABS composites were performed in accordance with the ASTM D790 standard using a universal testing machine (UTM, Shimadzu JP, AG-50kNX, Kyoto, Japan). The dimensions of bar-shaped specimen were 125 mm × 12.5 mm × 3 mm. The span-to-depth ratio was 32 and the span length between the two supports was 96 mm. A load cell of 50 kN and crosshead speed of 5.1 mm/min were used. The average values of flexural modulus and strength of each sample were obtained from 10 specimens.

#### 2.3.9. Impact Test

Izod impact tests were performed with neat ABS and single-sized and dual-sized carbon fiber/ABS composites according to the ASTM D256 standard using a pendulum-type impact tester (Tinius Olsen, Model 867, Horsham, PA, USA). The dimensions of each specimen were 63.5 mm in length, 12.5 mm in width, and 3 mm in thickness. Each specimen has a V-shaped notch (2.5 mm in depth) made using a notch cutter. The impact rate was 3.46 m/s. The impact distance was 610 mm. The impact energy of 12.66 J was used. The average Izod impact strength of each sample was obtained from 10 specimens.

## 3. Results and Discussion

### 3.1. Surface Topography Analysis of Carbon Fiber

**Figure 2:**
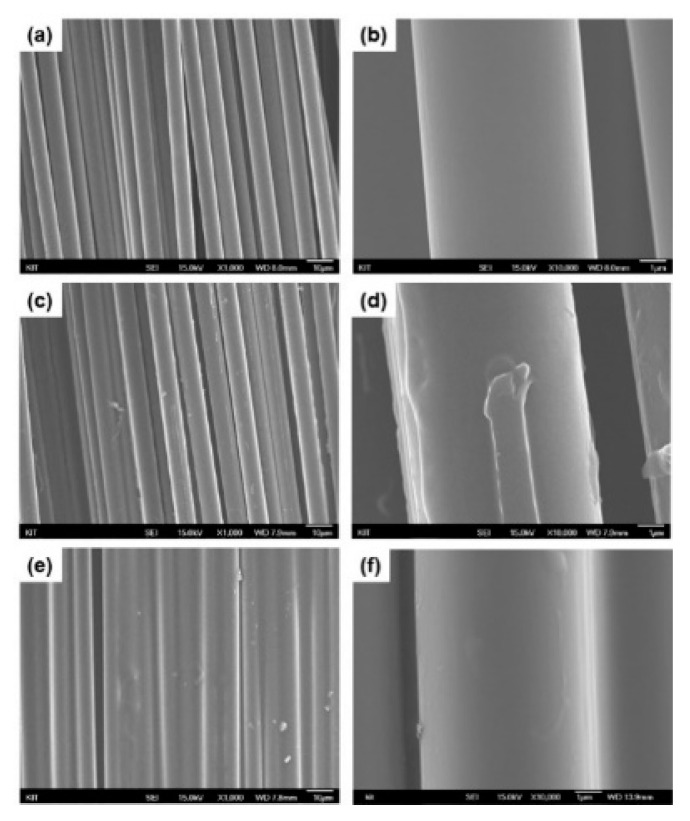
SEM micrographs (**a**,**c**,**e**: ×1000, **b**,**d**,**f**: ×10,000) showing the surfaces of (**a**,**b**) (epoxy) single-sized carbon fiber, (**c**,**d**) (epoxy + epoxy) dual-sized carbon fiber, and (**e**,**f**) (epoxy + PU) dual-sized carbon fiber.

Figure 2 displays the SEM images of single-sized and dual-sized carbon fiber surfaces at different magnifications. As seen from the carbon fiber single-sized with epoxy in Figure 2a,b, most of the individual carbon fiber filaments were seperated from each other. The fiber showed typical carbon fiber surface, as found in commercial PAN-based carbon fibers [41]. Figure 2c,d exhibit the surface topography observed with (epoxy + epoxy) dual-sized carbon fiber. The rough fiber surface formed by dual-sizing process was observed with carbon fiber filaments apart from each other.

Figure 2e,f represent the surface of (epoxy + PU) dual-sized carbon fiber. The fiber surface was distinguishable, compared to the single-sized and (epoxy + epoxy) dual-sized cases, indicating that many individual fiber filaments were stuck together, being ascribed to additional sizing with PU. The dual-sized carbon fiber surface became smooth because the surface was coated with the secondary sizing layer. It may be expected that strong hydrogen bonds formed between epoxy and PU [42] contributed to binding the filaments together during dual-sizing process. The additionally sized surface was clearly found in both dual-sized carbon fiber cases. It was thought that such a dual-sized surface may influence the interfacial characteristic between carbon fiber and polymer matrix, not only by the physical change of fiber surface, but also by the chemical interaction between the fiber surface and the polymer matrix [43], influencing the mechanical and thermal properties of resulting composites.

**Figure 3:**
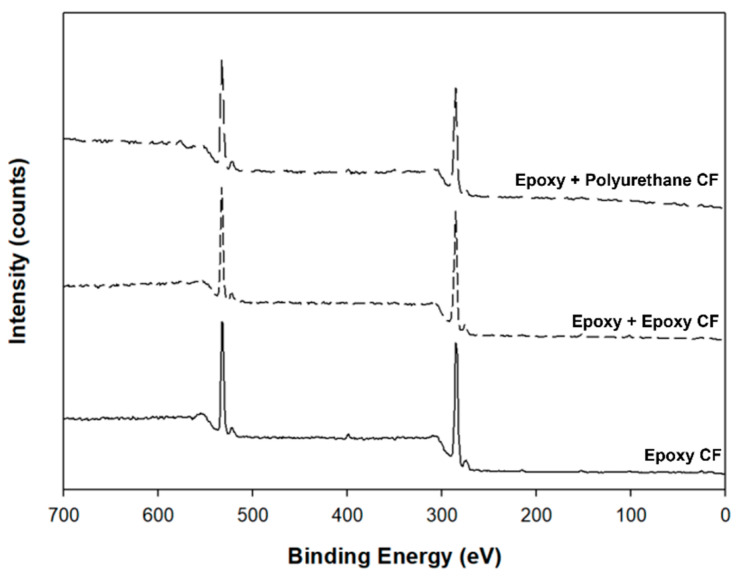
XPS survey scans of carbon fibers sized with epoxy, epoxy + epoxy, and epoxy + PU.

XPS is a surface-sensitive spectroscopic technique based on the photoelectric effect that can identify the chemical elements existing in a material as well as its chemical state. XPS is useful because it informs what elements are present on the surface and what other elements are bonded to them [44,45]. Figure 3 shows the XPS survey scans measured with single-sized and dual-sized carbon fibers. Three main characteristic peaks were due to the carbon element at about 285 eV, due to the oxygen element at about 533 eV, and due to the nitrogen element at about 399 eV. The peak areas are listed in Table 1. Compared to single-sized carbon fiber, the carbon contents were decreased, whereas the oxygen contents were increased in dual-sized carbon fibers, owing to additional sizing with either epoxy or PU. In single-sized carbon fiber, the nitrogen element existing on the surface was about 1.4%. This may be ascribed to the nitrogen component remaining in commercial PAN-based carbon fiber. In (epoxy + epoxy) dual-sized carbon fiber, the nitrogen element of about 0.4% was measured. Such a low nitrogen concentration can be explained by that the surface of commercial carbon fiber was covered by additional epoxy sizing so that the nitrogen element existing on the fiber surface was not fully detected. Another reason for this was that a very small amount of nitrogen, which originated from the amine-based hardener contained in epoxy resin, may be present on the dual-sized carbon fiber surface. The nitrogen concentration was increased to about 1.0% in (epoxy + PU) dual-sized carbon fiber. This was attributed to the nitrogen component containing in the isocyanate group (-NCO) of PU.

### 3.2. Fiber Length Distribution in Carbon Fiber/ABS Composites

**Figure 4:**
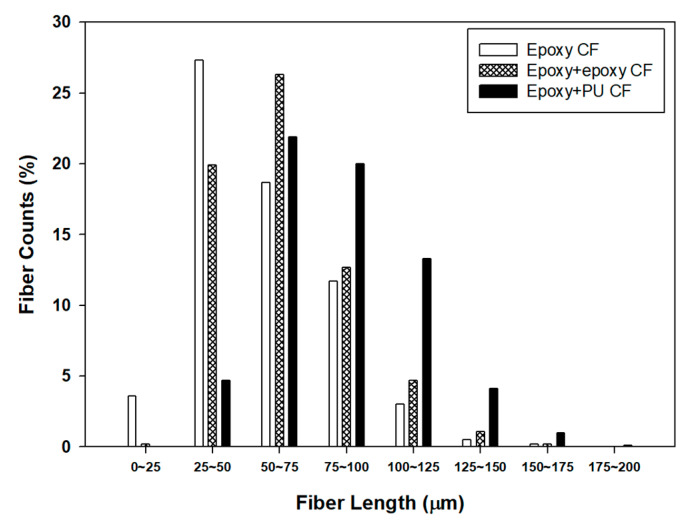
Fiber length distribution histogram for carbon fiber/ABS composites with (epoxy) single-sized and (epoxy + epoxy, epoxy + PU) dual-sized carbon fibers.

The dual-sizing effect on the length distribution of carbon fibers in carbon fiber/ABS composites prepared by extrusion and injection molding processes was investigated, as shown in Figure 4. The composites with (epoxy) single-sized carbon fiber exhibited the highest fiber counts in the fiber length range of 25–50 μm and relatively high populations in the range of 50–75 and 75–100 μm. The average carbon fiber length was about 57.1 μm. Meanwhile, the composites with either (epoxy + epoxy) or (epoxy + PU) dual-sized carbon fiber showed the average fiber length of about 64.8 and 85.0 μm, respectively. The result indicated that the composite with dual-sized carbon fiber had the higher fiber counts, and the average fiber length was longer than that with single-sized carbon fiber. In addition, the composite with (epoxy + PU) dual-sized carbon fiber had the higher fiber counts, and the average fiber length was longer than that with (epoxy + epoxy) dual-sized carbon fibers, showing an average fiber length in the range of 50–150 μm and even longer.

The fiber length distribution analysis can be explained by that chopped carbon fibers experienced shear forces occurring between the barrel and the screw in the twin-screw extruder during extrusion and injection processes, resulting in shortening of the fiber length [46]. As a result, the aspect ratio of carbon fiber in the composite was decreased with some variations. In general, sizing can contribute not only to protecting possible fiber damages occurring upon extrusion/injection processing but also to improving the interfacial bonding between the fiber and the matrix [26]. Kim et al. [47] reported that the use of sized carbon fiber resulted in a longer fiber length than that of unsized carbon fiber in carbon fiber/polycarbonate composites via the extrusion process. Accordingly, it can be said that dual sizing may play a positive role in reducing possible carbon fiber damage and shortening by shear forces occurring during extrusion and injection processes, giving rise to some benefits, compared to the single sizing case. The result also indicated that the fiber length distribution and the fiber aspect ratio strongly depended on the sizing level, implying a possible effect on the properties of relevant composites.

### 3.3. Thermal Stability of Composites

**Figure 5:**
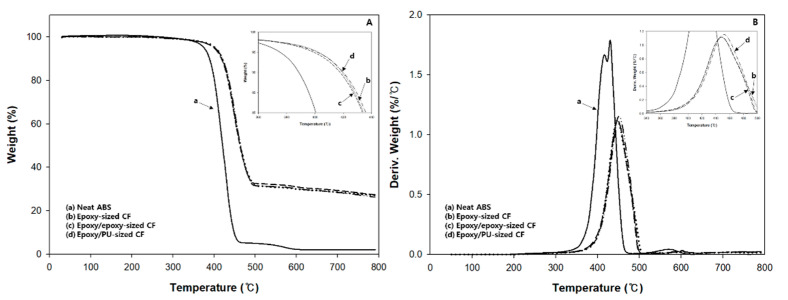
(**A**) TGA and (**B**) DTG curves measured for neat ABS and carbon fiber/ABS composites with (epoxy) single-sized and (epoxy + epoxy, epoxy + PU) dual-sized carbon fibers.

Figure 5 shows the thermal stability of neat ABS and carbon fiber/ABS composites with single-sized and dual-sized carbon fibers. In neat ABS, the initial weight loss began at about 250 °C and an abrupt weight loss occurred in the range of 340–460 °C due to thermal decomposition by the butadiene component at about 340 °C, by the styrene component at about 350 °C, and by the acrylonitrile component at about 400 °C, as similarly observed in other studies [48,49]. The weight loss in the range of 460–600 °C was due to decomposition of the aromatic compounds therein besides the main components in ABS resin.

The thermal stability of the composite with dual-sized carbon fiber was slightly higher than that with single-sized carbon fiber even though their overall weight loss behaviors were similar, as seen in the inserted curves of Figure 5A. All the composites showed an abrupt weight loss up to 450 °C with the initial weight loss at about 290 °C. Then, the weight was slowly decreased with temperature, indicating the residual weight of about 30% at about 790 °C due mainly to the carbon fiber therein. It turns out that the carbon fiber contents in the composite were about 30% by weight. In the derivative thermogravimetric (DTG) curves of Figure 5B, the maximum decomposition rate of neat ABS was highest at 430 °C. The temperature (452 °C) showing the maximum decomposition rate of the composite with (epoxy + PU) dual-sized carbon fiber was 4 °C higher than that (448 °C) with single-sized carbon fiber or with (epoxy + epoxy) dual-sized carbon fiber. The DTG result indicated that the (epoxy + PU) dual-sizing shifted the decomposition temperature higher, due to the increased fiber–matrix interaction in the composite.

### 3.4. Heat Deflection Temperature of Composites

**Figure 6:**
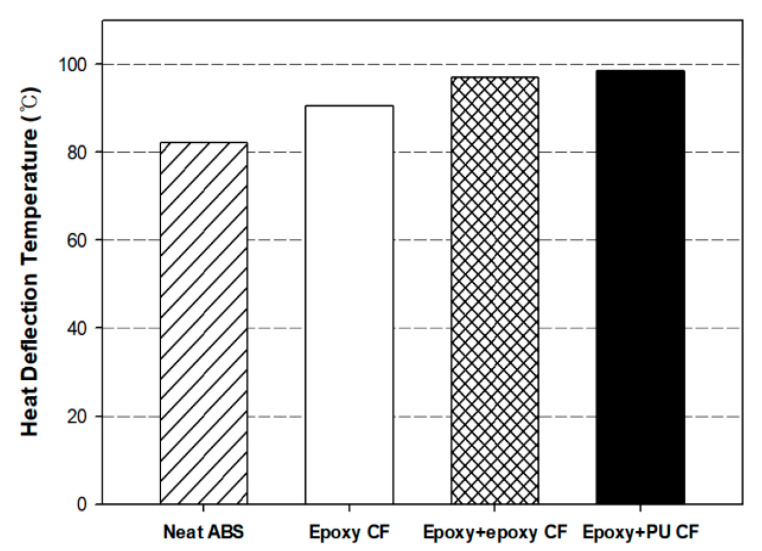
Heat deflection temperatures of neat ABS and carbon fiber/ABS composites with (epoxy) single-sized and (epoxy + epoxy, epoxy + PU) dual-sized carbon fibers.

Figure 6 represents the dual-sizing effect on the heat deflection temperature (HTT) of neat ABS and carbon fiber/ABS composites. The HDT of neat ABS resin was about 82 °C. The HDT of the composites with single-sized carbon fiber was 9 °C. The HDT values of the composites with dual-sized carbon fiber were 97 °C for the (epoxy + epoxy) sample and 99 °C for the (epoxy + PU) sample. The HDT of all the composites was 9–17 °C higher than that of neat ABS, obviously reflecting a reinforcing effect by carbon fiber. The HDT increase was due to the increase of load transferring ability in the composite under the test environment in a silicone oil bath.

The HDT of the composites with dual-sized carbon fiber was 6–8 °C higher than that of the composite with single-sized carbon fiber. It was noted that the composite with (epoxy + epoxy) dual-sized carbon fiber exhibited slightly higher HDT than that with (epoxy + PU) dual-sized carbon fiber. This can be explained by the fact that dual sizing resulted in stronger interfacial bonding between the individual carbon fiber and the ABS matrix than the single sizing case with epoxy only. The dual-sizing with both epoxy and PU played a beneficial role in increasing the interfacial bonding in comparison to the dual-sizing with epoxy and epoxy. In fact, while ABS pellets were melted and compounded with chopped carbon fibers during extrusion process, the additionally sized material contributed somewhat to binding the individual carbon fibers together. In addition, the additionally sized PU on the commercially epoxy-sized carbon fiber surface may give rise to increasing the interfacial bonding between the fiber and the matrix more effectively than the additionally sized epoxy, due to the increased chemical interaction [50].

### 3.5. Dynamic Mechanical Properties of Composites

Dynamic mechanical properties are very sensitive to molecular mobility change occurring with increasing temperature, particularly near the glass transition region. Therefore, the stiffness change of molecular chains is closely relevant to the storage modulus change, varying with temperature. The modulus is generally decreased with increasing temperature, profoundly decreasing above the glass transition temperature, due mainly to the increase of molecular motion.

**Figure 7:**
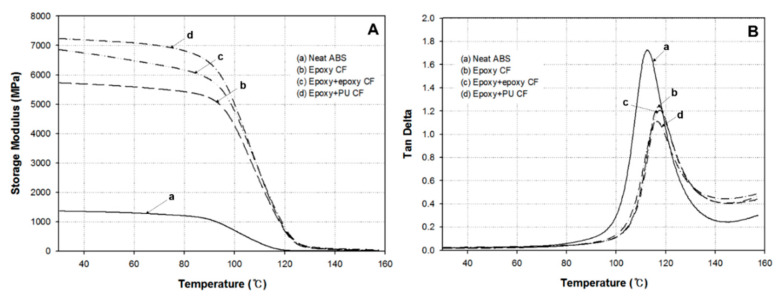
Variations of the (**A**) storage modulus and (**B**) tan δ of neat ABS and carbon fiber/ABS composites with (epoxy) single-sized and (epoxy + epoxy, epoxy + PU) dual-sized carbon fibers.

Figure 7 displays the variation of the (A) storage modulus and (B) tan δ of neat ABS and carbon fiber/ABS composites with single-sized and dual-sized carbon fibers, showing a dual-sizing effect on the dynamic mechanical properties of carbon fiber/ABS composites. As seen in Figure 7A, the storage modulus of neat ABS without reinforcing fibers was lowest, whereas the tan δ at the peak temperature was highest. Due to the reinforcing effect of carbon fiber, the storage modulus of the composite was highly increased, whereas the tan δ was decreased as well. The storage modulus of the composite with dual-sized carbon fiber was higher than that with single-sized carbon fiber. The storage modulus of the composite with (epoxy + PU) dual-sized carbon fiber was even higher than that with (epoxy + epoxy) dual-sized carbon fiber. The storage moduli of the composites at 30 °C were 5.7 GPa with single-sized carbon fiber, 6.9 GPa with (epoxy + epoxy) dual-sized carbon fiber, and 7.2 GPa with (epoxy + PU) dual-sized carbon fiber. The composite with (epoxy + PU) dual-sized carbon fiber exhibited the highest modulus in the measured temperature range. The storage modulus was largely decreased beyond about 90 °C. This can be explained by the increased molecular motion in the ABS matrix with increasing temperature, particularly, due mainly to the molecular mobility of the butadiene component in the low temperature region and the acrylonitrile and styrene components in the high temperature region.

In the composite with dual-sized carbon fiber, the molecular mobility of ABS resin surrounding the individual fibers was more restricted than in the composite with the single-sized one. As a result, the storage modulus was increased. In fiber-reinforced polymer matrix composites, the storage modulus is relevant to the interfacial bonding between the fiber and the polymer matrix therein, influencing their stiffness and modulus [51]. Moreover, the interfacial bonding may be influenced by the chemical constituents existing on the fiber surface, being increased by the strong interaction between the fiber and the matrix consisting of a composite material [52]. Accordingly, it may be said that one possible reason for the highest storage modulus of the composite with (epoxy + PU) dual-sized carbon fiber was that its interfacial bonding between the carbon fiber and the ABS matrix was best, compared to the single-sized and (epoxy + epoxy) dual-sized cases, as supported by the mechanical data and the fracture surface analysis of resulting composites hereinafter.

tan δ is closely related to damping characteristic of a material. In general, the higher tan δ value indicates the higher damping and energy dissipating capability of a polymeric material [53]. As seen in Figure 7B, the tan δ peak height was highest with neat ABS, and lowest with the composite having (epoxy + PU) dual-sized carbon fiber. Owing to the fiber reinforcing effect, the peak height was obviously decreased. The incorporation of carbon fiber into neat ABS restricted the mobility of ABS molecular chains, resulting in the decrease of loss modulus, leading to the reduction of tan δ peak height in the composite [52,54,55]. The peak temperature of neat ABS was 112 °C and it was shifted about 5 °C to a higher temperature in the composite. Moreover, the interfacial bonding between the fiber and the matrix influenced the tan δ variation, as described earlier [56].

### 3.6. Tensile Properties of Composites

**Figure 8:**
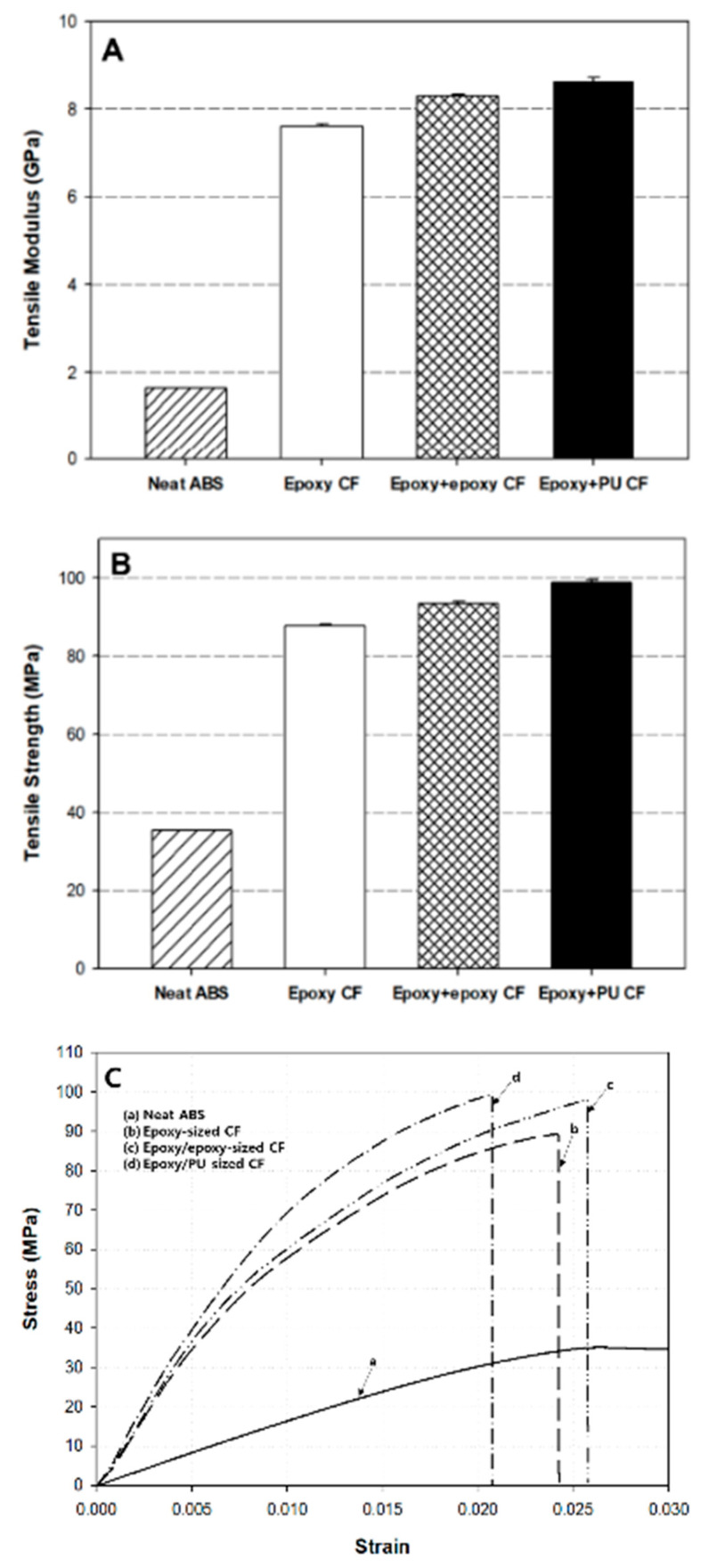
Comparisons of the (**A**) tensile modulus, (**B**) strength, and (**C**) stress-strain curves of neat ABS and carbon fiber/ABS composites with (epoxy) single-sized and (epoxy + epoxy, epoxy + PU) dual-sized carbon fibers.

Figure 8 represents the dual-sizing effect on the (A) tensile modulus, (B) tensile strength, and (C) stress–strain curves of carbon fiber/ABS composites. Overall, the variation tendency of the tensile modulus and strength was similar. Neat ABS exhibited the lowest tensile modulus and strength. The tensile modulus and strength of the composite were markedly increased due surely to the reinforcing effect by carbon fiber therein. The modulus was increased by 4.5–5 times and the strength was increased by 2.4–2.8 times, in comparison to neat ABS. The tensile modulus and strength of the composite with dual-sized carbon fiber were higher than those with single-sized carbon fiber. The composite with (epoxy + PU) dual-sized carbon fiber exhibited higher tensile modulus and strength than that with (epoxy + epoxy) dual-sized carbon fiber. In a composite material, the tensile properties strongly depend not only on the fiber/resin ratio and the fiber aspect ratio, but also on the fiber–matrix interfacial bonding, processing method, etc. Better adhesion at the interface between fiber and matrix can efficiently transfer the external mechanical load to the surrounding fiber and matrix. Sizing plays an important role in influencing the interfacial adhesion [20,57]. The tensile result revealed that the incorporation of dual-sized carbon fiber into the ABS matrix was more effective to increase the mechanical properties of resulting composites than that of single-sized carbon fiber into the matrix. In addition, the property improvement by the dual-sizing effect with epoxy and PU was higher than that by the dual-sizing effect with epoxy and epoxy. It was believed that the enhanced bonding at the interface between the carbon fiber and the ABS matrix by dual-sizing with epoxy and PU contributed to increasing the tensile modulus and strength.

From a mechanical viewpoint, tensile modulus is closely related to storage modulus because both are significantly affected by a material’s stiffness. Comparing the variations of the tensile modulus and the storage modulus above-mentioned, they agreed well with each other. The ductility of the composite was reduced by incorporating carbon fiber into the ABS matrix due to the stiffening effect, resulting in the decreased strain and the increased modulus. In the stress–strain (S-S) curves of Figure 8C, neat ABS exhibited more than 3% elongation. The stress and the slope of the S-S curve measured for the composites (c and d) with dual-sized carbon fiber were higher than those (b) with single-sized carbon fiber. The slope of the S-S curve reflected that the modulus of the composite with (epoxy + PU) dual-sized carbon fiber was higher than that with (epoxy + epoxy) dual-sized carbon fiber. The elongation at break of the single-sized composite was comparable to the (epoxy + epoxy) dual-sized case, whereas the composite with (epoxy + PU) dual-sized carbon fiber exhibited the lowest elongation at break than other composite cases.

### 3.7. Flexural Properties of Composites

**Figure 9:**
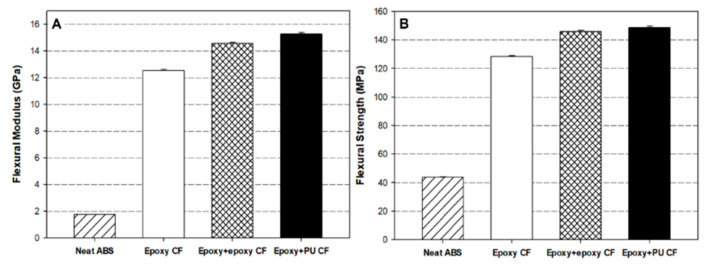
Comparisons of the (**A**) flexural modulus and (**B**) strength of neat ABS and carbon fiber/ABS composites with (epoxy) single-sized and (epoxy + epoxy, epoxy + PU) dual-sized carbon fibers.

Figure 9 displays the dual-sizing effect on the flexural modulus (A) and strength (B) of carbon fiber/ABS composites. The variation of the flexural properties was similar to that of the tensile properties. Neat ABS exhibited the lowest flexural modulus and strength. Owing to the carbon fiber reinforcement, the flexural modulus of the composites was remarkably enhanced by 7–9 times from 1.8 GPa to 12.5–15.4 GPa and the flexural strength was highly increased by 3–3.5 times from 43 MPa to 129–150 MPa, depending on the sizing type described above. With a close inspection, it was revealed that the flexural property improvement was more pronounced than the tensile property improvement with the corresponding fiber loading and composite fabrication process. This can be explained by that the tensile properties of a fiber-reinforced composite strongly depend on the fiber alignment along with the longitudinal direction of a specimen, whereas the flexural properties are mainly influenced by the fiber alignment through the thickness direction of a specimen under three-point flexural load. The carbon fibers with different fiber length distributions, as described in Figure 4, were located along the through-the-thickness direction as well as the longitudinal direction of each composite specimen. During the tensile test, the carbon fibers existing in the longitudinal direction of the specimen were obviously responsible for the increased tensile property, but the carbon fibers existing in the through-the-thickness direction did not influence the property as strongly as in the longitudinal direction. On the other hand, in the case of the three-point flexural test, all the carbon fibers in any directions may contribute to increasing the flexural modulus and strength because the compressive force acting in the through-the-thickness direction and the tensile force acting in the longitudinal direction are responsible for the flexural property. Therefore, it may be said that the interfacial bonding between the fiber and the matrix was more responsible for the flexural property than for the tensile property, indicating that the flexural test was useful to study the interfacial behavior of fiber-reinforced composites [58].

The flexural modulus and strength of the composite with dual-sized carbon fiber were higher than those with single-sized carbon fiber. The composite with (epoxy + PU) dual-sized carbon fiber showed the highest flexural modulus and strength. The composite with (epoxy + PU) dual-sized carbon fiber resulted in the flexural modulus and strength higher than that with (epoxy + epoxy) dual-sized carbon fiber. It was emphasized that the interfacial bonding between the carbon fiber and the ABS matrix was higher with (epoxy + PU) dual-sized carbon fiber than with (epoxy + epoxy) dual-sized one.

### 3.8. Izod Impact Strength of Composites

**Figure 10:**
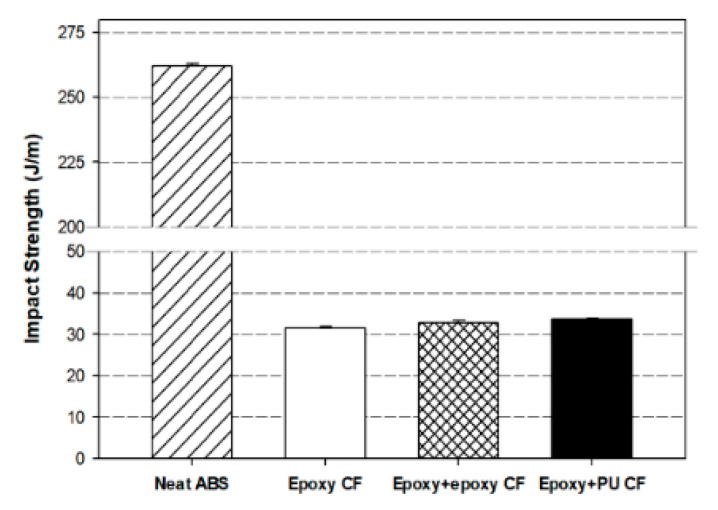
Izod impact strengths of neat ABS and carbon fiber/ABS composites with (epoxy) single-sized and (epoxy + epoxy, epoxy + PU) dual-sized carbon fibers.

Figure 10 compares the Izod impact strengths measured with neat ABS and carbon fiber/ABS composites with different carbon fiber sizing materials. Among the samples, neat ABS exhibited the highest impact strength of 262 J/m, as expected. Such high impact strength was ascribed to the intrinsic character of ABS resin, which can absorb the external impact energy owing to the presence of the butadiene component in ABS resin. The impact strength of the composite was much lower than that of neat ABS due to the presence of brittle carbon fibers in the ductile ABS matrix [35].

The decreased impact strength of the composite with single-sized carbon fiber was slightly increased by the dual-sizing effect. The composite with (epoxy + PU) dual-sized carbon fiber exhibited the impact strength higher than that with (epoxy + epoxy) dual-sized one. As indicated in Figure 4, the composite having the carbon fiber dual-sized with epoxy and PU exhibited a longer fiber length than that dual-sized with epoxy and epoxy. It was strongly indicated that the longer carbon fiber more effectively contributed to resisting the external impact energy than the shorter fiber in the composite, distributing the external impact energy from the matrix to the neighboring fiber [59]. In addition, the increased interfacial bonding in the composite with (epoxy + PU) dual-sized carbon fiber contributed to transferring the impact energy to the individual fibers and the surrounding matrix.

### 3.9. Fracture Surfaces of Composites

**Figure 11:**
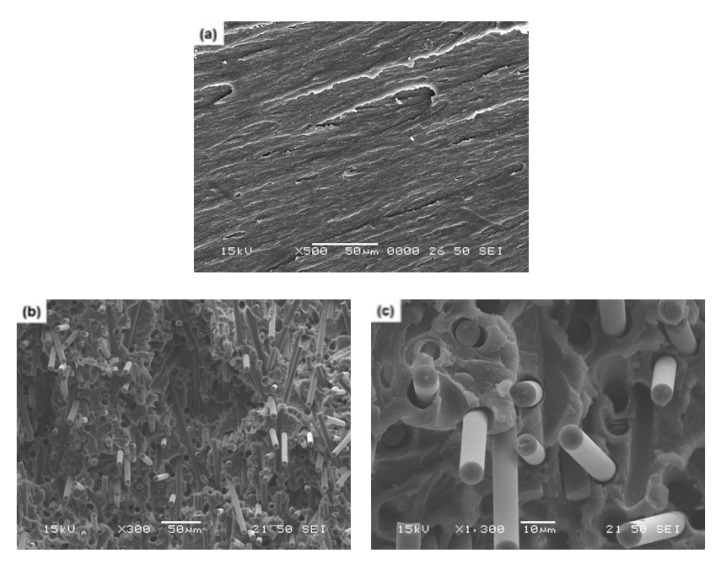
SEM micrographs (**a**,**b**: ×500, **c**: ×1300) showing the fracture surfaces of (**a**) neat ABS and (**b**,**c**) carbon fiber/ABS composites with (epoxy) single-sized carbon fiber.

In a fiber-reinforced composite material, the fracture surface is a good indication to analyze the interfacial behavior between the fiber and the surrounding matrix and further to foresee the mechanical properties therefrom. Upon fracture, such microscopic behavior may influence the absorption and dissipation of externally applied impact energy, depending on the degree of interfacial adhesion between the fiber and the matrix [60]. Figure 11 displays the fracture surface topography observed after impact testing for (a) neat ABS and (b) carbon fiber/ABS composites with single-sized carbon fiber. Neat ABS exhibited a typical ductile fracture pattern, as found with the thermoplastic matrix. As can be seen from the SEM images of the composite, fiber pull-out, fiber breakage, and debonding between the fiber and the matrix were observed. The carbon fiber surfaces pulled out from the matrix remained clean, showing some gaps by debonding between the fiber and the matrix. The length of the broken fibers was relatively long. This indicated that the interfacial bonding between the carbon fiber and the ABS matrix was poor [52]. Such poor bonding resulted in decreased mechanical properties, as described with the mechanical result earlier.

**Figure 12:**
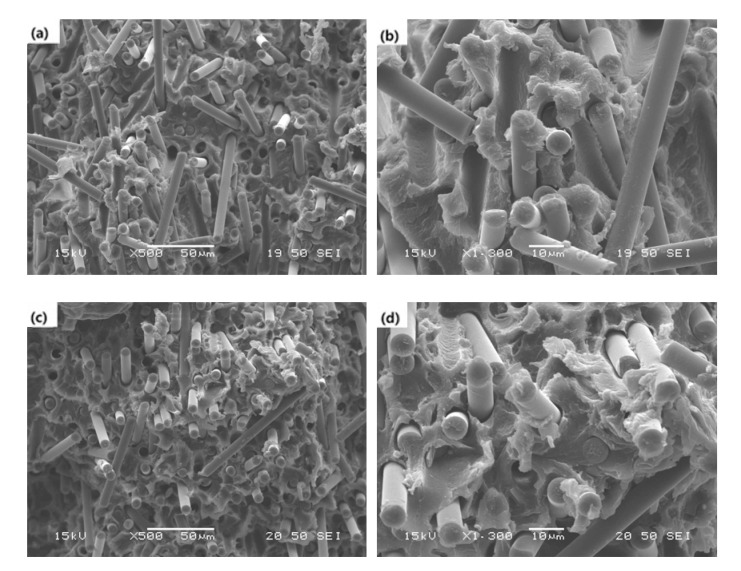
SEM micrographs (**a**,**c**: ×500, **b**,**d**: ×1300) showing the fracture surfaces of carbon fiber/ABS composites with (**a**,**b**) (epoxy + epoxy) dual-sized carbon fiber and (**c**,**d**) (epoxy + PU) dual-sized carbon fiber.

Figure 12 displays SEM micrographs observed from the fracture surface of carbon fiber/ABS composites with (a,b) (epoxy + epoxy) dual-sized, and (c,d) (epoxy + PU) dual-sized carbon fibers. The fracture surfaces were quite distinct from the surface topography seen in Figure 11. It seemed that the interfacial bonding between the fiber and the matrix in the dual-sized case was better than that in the single-sized one. In addition, the interfacial bonding with (epoxy + PU) dual-sized carbon fiber was better than that with the (epoxy + epoxy) dual-sized counterpart. The good interfacial bonding was obviously found from the less pulled-out fibers, the shorter broken fibers, the matrix stuck on the pulled-out fiber surface, and the reduced gaps between the fiber and the matrix [61]. It was strongly indicated that the interfacial behavior directly influenced the thermal, mechanical, and impact properties, as described earlier. The better the interfacial adhesion between the fiber and the matrix, the higher the properties of a composite material. The fracture surface topography of the composites was responsible for the mechanical and impact properties of the composites, qualitatively supporting that the tensile and flexural modulus and strength and the Izod impact strength of the composite with dual-sized carbon fiber were higher than those with the single-sized counterpart. Moreover, the composite with (epoxy + PU) dual-sized carbon fiber had higher tensile, flexural, and impact properties than that of the composite with (epoxy + epoxy) dual-sized carbon fiber.

As indicated earlier, the oxygen concentration on the dual-sized carbon fiber surface was higher than that on the single-sized carbon fiber surface. The oxygen concentration was higher with (epoxy + PU) dual-sized carbon fiber than with (epoxy + epoxy) dual-sized carbon fiber. As a result, it played a role not only in enhancing the fiber–matrix interfacial bonding, but also in increasing the thermal and mechanical properties of resulting composites.

## 4. Conclusions

Dual-sized carbon fiber exhibited an oxygen content 3–5% higher than single-sized carbon fiber, indicating the highest oxygen with the (epoxy + PU) dual-sized carbon fiber. In addition, the composite with dual-sized carbon fiber showed a longer fiber length than that with single-sized carbon fiber. The composite with (epoxy + PU) dual-sized carbon fiber showed a longer fiber length than that with the (epoxy + epoxy) dual-sized counterpart. The results evince a role not only in enhancing the interfacial bonding between the carbon fiber and the ABS matrix, but also in increasing the thermal, mechanical, and impact properties of resulting composites.

The HDT and the storage modulus of the composite with dual-sized carbon fiber were significantly higher than those with single-sized carbon fiber. The composite with (epoxy + PU) dual-sized carbon fiber exhibited the highest HDT and storage modulus relative to the (epoxy + epoxy) dual-sized case. The tensile and flexural properties of neat ABS were markedly increased by reinforcing with single-sized carbon fiber and further increased by reinforcing with dual-sized carbon fiber. The property enhancement effect was highest with (epoxy + PU) dual-sized carbon fiber. It was ascribed to the enhanced interfacial bonding as well as to the increased aspect ratio of carbon fiber in the composite by the dual-sizing effect using both epoxy and PU together. The result indicated that the flexural property of carbon fiber/ABS composite was more pronouncedly increased than the tensile property, reflecting that the enhanced interfacial bonding between the carbon fiber and the ABS matrix was more responsible for it.

It can be stated that the enhanced interfacial bonding, which was established by dual-sized carbon fiber reinforcement, was responsible for the improvement of the thermal, mechanical, and impact properties of carbon fiber/ABS composites, being supported by the fiber length distribution analysis and the fracture surface topography.

## Figures and Tables

**Table 1:** The atomic contents existing on the surfaces of (epoxy) single-sized and (epoxy + epoxy) and (epoxy + PU) dual-sized carbon fibers measured by XPS.

Carbon Fiber Sizing Type	Carbon Atomic C1s (%)	Oxygen Atomic O1s (%)	Nitrogen Atomic N1s (%)
Epoxy	77.7	20.9	1.4
Epoxy + epoxy	77.1	22.5	0.4
Epoxy + PU	74.0	25.0	1.0

## Data Availability

The data presented in this study are available on request from the corresponding author.
